# Representação e representatividade nos conselhos de saúde: entre a formalização e a legitimidade participativa

**DOI:** 10.1590/0102-311XPT115524

**Published:** 2025-02-07

**Authors:** Edna Moreira Barros, José Patrício, Luzia Célia Batista Soares, Mauro Serapioni

**Affiliations:** 1 Instituto Multidisciplinar de Saúde, Universidade Federal da Bahia, Vitória da Conquista, Brasil.; 2 Centro de Estudos Sociais, Universidade de Coimbra, Coimbra, Portugal.

**Keywords:** Conselhos de Saúde, Participação Social, Representação dos Pacientes, Políticas de Saúde, Sistema Único de Saúde, Health Councils, Social Participation, Patient Representative, Health Policy, Unified Health System, Consejos de Salud, Participación Social, Representantes de los Pacientes, Política de Salud, Sistema Único de Salud

## Abstract

A atuação dos representantes sociais exerce grande influência sobre o desempenho dos conselhos de saúde e a qualidade da gestão participativa no Sistema Único de Saúde. Este artigo objetivou analisar os mecanismos de representação e as formas de representatividade no âmbito de conselhos de saúde em municípios de diferentes portes populacionais. Trata-se de um estudo de casos múltiplos realizado em Vitória da Conquista, Guanambi e Urandi, na Bahia, Brasil. Os dados e informações foram obtidos por meio da análise de documentos, observação de reuniões plenárias e entrevistas com 30 conselheiros de saúde. Para a análise dos dados, utilizou-se uma matriz teórica estruturada em cinco dimensões: processo de escolha dos representantes; relação entre representantes e representados; tipos de interesse representados; critérios para definição de posicionamentos; e prática participativa. Os resultados demonstraram o predomínio de critérios não eleitorais para a escolha dos representantes. A relação entre representantes e representados é marcada pelo isolamento do representante e reduzida prestação de contas. Predominou a consciência individual na definição de posicionamentos por parte dos conselheiros. A representação nos conselhos de saúde também se mostrou influenciada pelo porte populacional dos municípios. No município de menor população, evidenciou-se consistentes fragilidades para o exercício da representação. Os resultados demonstraram insuficiências do processo representativo. Ressalta-se a necessidade de aproximação entre representantes e representados e a importância da valorização do conhecimento experiencial como elemento capaz de conceder legitimidade à representação.

## Abstract (en)

The action of social representatives greatly influences the performance of health councils and the quality of participatory management in the Brazilian Unified National Health System. This study aimed to analyze the mechanisms of representation and forms of representativeness within health councils in municipalities of different population sizes. This multiple case study was carried out in Vitória da Conquista, Guanambi, and Urandi, in Bahia State, Brazil. Data and information were obtained by document analysis, observation of plenary meetings, and interviews with 30 health board members. A theoretical matrix structured in five dimensions was used for data analysis: the process to choose representatives; relationships between representatives and those who are represented; types of represented interests; criteria to define positions; and participatory practice. Results showed the predominance of non-electoral criteria to choose representatives. The relationship between representatives and those who are represented shows the former’s isolation and reduced accountability. Individual conscience predominated as the board defined positions. The population size of the municipalities also influenced representation in health councils as that with the smallest population evinced consistent weaknesses for the exercise of representation. Results showed insufficiencies in the representative process. This study stresses the need to draw representatives and those they represent near each other and the importance of valuing experiential knowledge as an element that can grant legitimacy to representation.

## Resumen

La acción de los representantes sociales tiene una gran influencia en el desempeño de los consejos de salud y en la calidad de la gestión participativa en el Sistema Único de Salud. Este artículo tuvo como objetivo analizar los mecanismos de representación y las formas de representatividad en el ámbito de los consejos de salud en municipios de diferentes tamaños de la población. Este es un estudio de caso múltiple realizado en Vitória da Conquista, Guanambi y Urandi, en Bahía, Brasil. Los datos y la información se obtuvieron del análisis de documentos, observación de reuniones plenarias y entrevistas con 30 consejeros de salud. Para el análisis de datos, se utilizó una matriz teórica estructurada en cinco dimensiones: proceso de elección de representantes; relación entre representantes y representados; tipos de interés representados; criterios para definir posiciones; y práctica participativa. Los resultados demostraron el predominio de criterios no electorales para la elección de los representantes. La relación entre los representantes y los representados estuvo marcada por el aislamiento del representante y la reducción de la responsabilidad. Predominó la conciencia individual en la definición de cargos por parte de los consejeros. La representación en los consejos de salud también se vio influenciada por el tamaño de la población de los municipios. En el municipio con menor población, hubo debilidades consistentes para el ejercicio de la representación. Los resultados demostraron deficiencias del proceso representativo. Cabe destacar la necesidad de acercamiento entre los representantes y los representados, y la importancia de valorar la experiencia como un elemento capaz de otorgar legitimidad a la representación.

## Introdução

A participação social exercida no âmbito dos conselhos de saúde apresenta diversos desafios. Entre estes, destaca-se o tema da representatividade dos participantes. A representação tem sido apontada como aspecto crítico das experiências de participação social ^1^ ^,^ ^2^ e fator limitador para o desempenho dos conselhos de saúde ^3^. Nesse contexto, é importante observar que a representação em instâncias participativas difere consideravelmente dos aspectos de legitimação da representação eleitoral clássica ^4^. Diante da ampliação dos mecanismos participativos nos sistemas de saúde, ampliou-se também a atuação dos representantes sociais e com isso a necessidade de aprofundamento dos aspectos inerentes à representatividade dos participantes e a legitimidade dos interesses representados.

Representar significa fazer presente alguém ou o interesse de outra pessoa mediante a figura de um intermediário ^5^. Nesse sentido, três aspectos se apresentam como essenciais: quem representa, quem é representado e qual interesse se representa. No que se refere à legitimidade, a teoria clássica da representação sinaliza a autorização, a identidade e a prestação de contas como elementos centrais da representação ^6^. Diante do pressuposto da autorização, a eleição assumiu centralidade no processo de constituição da representação. Todavia, com a ampliação dos espaços democráticos de participação, a representação eleitoral quantitativa passou a ser questionada como principal elemento que confere legitimidade à representação. De acordo com Loureiro ^7^, as eleições são instrumentos insuficientes de expressão de preferências, de responsividade e de representatividade.

Existem diferentes denominações e classificações de representação. Uma categorização clássica e amplamente difundida é a realizada por Pitkin ^6^. A autora considera quatro tipos de representação: (1) a formalística, centrada nos arranjos procedimentais de como os representantes são autorizados a agir em nome de outro; (2) a descritiva, refere-se à maneira pelas quais um representante se assemelha aos representados, diz respeito ao pertencimento e identidade; (3) a simbólica, relacionada a como os representantes utilizam significados, atitudes e crenças de forma simbólica, diz respeito ao significado que um representante tem diante dos representados; e (4) a substantiva, refere-se à qualidade empírica da representação e considera as ações praticadas pelo representante em nome do representado. Outras classificações que agrupam a representação em quantitativa e qualitativa ^5^ ou eleitoral e não eleitoral ^8^ buscam contextualizar que a representação não pode ser reduzida aos mecanismos formais de escolha.

No cenário internacional, a representatividade nos espaços de participação em saúde é permeada por muitos desafios. Estudos demonstram que a representação dos usuários nem sempre é capaz de vocalizar as necessidades de saúde de todos os segmentos da população, em especial daqueles mais desfavorecidos ^9^ ^,^ ^10^. Os Conselhos Comunitários de Saúde, no Serviço Nacional de Saúde (NHS; *National Health Service*) da Grã-Bretanha, têm sido criticados por não serem representativos de alguns grupos sociais, como pessoas com deficiência, com problemas de saúde mental, imigrantes e pessoas de baixa renda ^11^. No caso holandês, van de Bovenkamp & Vollaard ^12^ criticam a frágil interlocução e a distância entre os participantes ativos e aqueles que afirmam representar. Ressalta-se ainda a tendência de profissionais de saúde e gestores questionarem a representatividade dos usuários envolvidos em processos participativos como uma manobra para defender as relações de poder existentes e o controle dos processos de tomada de decisão ^13^. Muitos estudiosos também questionam a representatividade das associações de pacientes e usuários, nem sempre capazes de envolver certos grupos sociais, devido a dificuldades de comunicação, condições sociais e barreiras econômicas e culturais ^14^ ^,^ ^15^.

No Brasil, a participação da comunidade é uma diretriz do Sistema Único de Saúde (SUS) e os conselhos de saúde foram institucionalizados como parte da estrutura organizacional do sistema ^16^. Esses conselhos são compostos por representações de usuários, profissionais de saúde, prestadores de serviço e gestores. Trata-se de um mecanismo inovador e de ampliação da democracia, por possibilitar a inserção de novos atores à cena política ^17^. Desse modo, a ideia motora da democratização no SUS é que os conselhos de saúde possam funcionar como um canal de efetivação das demandas sociais por direitos, de construção coletiva das políticas de saúde e de formação de sujeitos políticos ^18^.

A *Resolução nº 453/2012* do Conselho Nacional de Saúde ^19^ estabelece os critérios e mecanismos da representação nos conselhos de saúde de forma a garantir a representatividade dos diversos segmentos sociais. A resolução indica quais segmentos podem compor a representação de usuários e profissionais e recomenda que os representantes sejam eleitos em fóruns especialmente convocados para esse fim ^20^.

Mesmo com tal regulamentação, ainda existem condições adversas que dificultam a garantia da representatividade nos conselhos de saúde e, assim, comprometem a gestão participativa no SUS. Entre os fatores limitadores, revela-se a fragilidade da relação entre representantes e representados. O distanciamento do representante das suas bases compromete a identificação de preferências e necessidades, e inviabiliza os mecanismos de prestação de contas e acompanhamento da atuação do representante ^1^. Diante desse contexto, Miguel ^21^ chama atenção sobre o deslocamento das relações representante-base para a relação representante-representante. O autor ressalta situações de exercício da representação por pessoas que não estabelecem qualquer interlocução válida com aqueles pelos quais diz falar, mas que é admitido como interlocutor legítimo por outras pessoas ou grupos que já ocupam posição nos espaços decisórios.

Emergem ainda como problemas da representação nos conselhos de saúde a falta de clareza de escolha dos representantes ^20^, a burocratização e tecnificação da participação ^17^, a profissionalização dos representantes ^3^, a desmobilização social e a falta de interesse na participação ^22^, a defesa de interesses pessoais em detrimento dos interesses dos representados ^23^, entre outros fatores. Apesar dos problemas evidenciados na literatura, cabe destacar que os conselhos de saúde apresentam desempenho heterogêneo e o exercício da representação é influenciado por condicionantes de diversas ordens. Nesse contexto, a influência da dimensão populacional dos municípios no exercício da representação nos conselhos de saúde é pouco conhecida.

O objetivo deste estudo foi analisar os mecanismos de representação e as formas de representatividade no âmbito de conselhos municipais de saúde (CMS) em municípios de diferentes portes populacionais.

## Métodos

### Delineamento do estudo

Este é um estudo de casos múltiplos com níveis de análise imbricados ^24^ e abordagem qualitativa. Os casos foram definidos com os CMS, enquanto instâncias participativas e de exercício da representação no SUS, em cidades de diferentes portes populacionais.

### Campo de investigação

O estudo foi realizado em três municípios da região sudoeste da Bahia, Brasil: Vitória da Conquista, Guanambi e Urandi. Estes são classificados, respectivamente como de grande, médio e pequeno porte. Adotou-se a classificação de Calvo et al. ^25^ que consideram grande porte as cidades com mais de 100 mil habitantes, médio porte aquelas com população entre 25 mil e 100 mil habitantes e pequeno porte as cidades com menos de 25 mil habitantes. Buscou-se, assim, averiguar como as diferentes realidades demográficas exercem influência no exercício da representação no âmbito dos conselhos de saúde.

Vitória da Conquista tem uma população de 370.879 habitantes e constitui-se como terceiro município mais populoso da Bahia ^26^. Tem ampla rede de serviços de saúde, é sede da Macrorregião de Saúde do Sudoeste da Bahia e se estabelece como referência na prestação de serviços de saúde para 74 municípios ^27^. O CMS é composto por 24 conselheiros de saúde titulares, tem sede própria, infraestrutura adequada e dispõe de secretaria executiva ^28^.

O Município de Guanambi tem população de 87.817 habitantes ^26^ e é sede de microrregião de saúde com 22 municípios ^27^. O CMS é composto por 16 conselheiros titulares, não tem sede própria, dispõe de secretaria executiva e a infraestrutura conta com computador com acesso à internet ^28^.

Urandi possui população de 15.355 pessoas ^26^ e integra a região de saúde de Guanambi ^27^. A composição do CMS é de 16 conselheiros titulares. O conselho não possui secretaria executiva e tem infraestrutura insuficiente, sem local fixo de funcionamento ^28^.

### Fonte de dados e participantes

Os dados foram coletados por meio de análise documental, observação participante e entrevistas semiestruturadas, no período de agosto de 2022 a junho de 2023. A análise documental contemplou os seguintes documentos: atas dos conselhos municipais de saúde dos anos de 2019 a 2021; Regimentos dos CMS; Planos Municipais de Saúde; Relatórios de Gestão; e Decretos, Portarias e Leis Municipais que tinham relação com a participação social.

A observação participante foi realizada durante as reuniões em plenárias dos CMS dos três municípios e registrada em diário de campo. Utilizou-se um roteiro para guiar a observação que contemplava os seguintes itens: identificação dos conselheiros e segmentos a que pertenciam; dinâmica da reunião; possibilidade de livre expressão dos conselheiros; poder de agenda dos representantes sociais; relações e articulações entre os representantes; e práticas participativas exercidas.

Foram entrevistados 30 conselheiros de saúde. Conforme apresentado na Tabela 1, em cada município entrevistou-se quatro representantes usuários, dois profissionais de saúde, dois gestores e dois prestadores. Partiu-se das análises das atas e da observação das reuniões para escolha dos participantes. Adotou-se o critério intencional determinado pelo protagonismo dos conselheiros e seus posicionamentos durante as reuniões. Como critérios de exclusão adotou-se ter sido conselheiro de saúde por período inferior a seis meses. O roteiro das entrevistas contemplou questões sobre a influência dos representantes dos usuários, a possibilidade de usuários inserirem temas na pauta, a dinâmica do processo deliberativo, o processo de escolha para atuar como conselheiro de saúde e as relações estabelecidas com os representados e as entidades. O tempo médio de cada entrevista foi de 69 minutos, com um total de 2.075 minutos de gravação.

**Tabela 1:** Participantes do estudo por segmento. Vitória da Conquista, Guanambi e Urandi, Bahia, Brasil, 2023.

Conselheiros entrevistadas	Vitória da Conquista	Guanambi	Urandi
Usuários	4	4	4
Profissionais de saúde	2	2	2
Gestores	2	2	2
Prestadores	2	2	2
Total	10	10	10

Fonte: elaboração própria, a partir dos dados do estudo.

### Procedimentos analíticos

Utilizou-se uma matriz de análise fundamentada em dimensões inerentes à representação e representatividade das instâncias de participação social. O desenvolvimento da matriz considerou as diretrizes do SUS e as peculiaridades do exercício da representação no âmbito dos CMS. No Quadro 1, estão dispostas as cinco dimensões e indicadores da matriz analítica.

**Quadro 1:** Matriz de análise referente à representação comunitária e à representatividade nos conselhos municipais de saúde.

DIMENSÃO	COMPONENTES
Processo de escolha de entidades e representantes	Método de escolha das entidades Métodos de escolha dos representantes
Relação entre representantes e representados	Atuação isolada do representante Acompanhamento pela entidade e Indicação de posicionamento Articulação com integrantes dos segmentos representados
Tipos de interesses representados	Interesses particulares Interesses corporativos Interesses amplos sobre saúde
Critérios para definição de posicionamento e opiniões	Consciência individual Articulação e formação de interesses com outros conselheiros Orientação e articulação com a entidade representada
Prática participativa do representante	Votante Reivindicativo Propositivo/participativo

Fonte: elaboração própria, fundamentada nos pressupostos da representação em instâncias participativas ^4^ ^,^ ^6^ ^,^ ^8^ ^,^ ^22^ ^,^ ^30^ ^,^ ^32^ ^,^ ^33^ ^,^ ^41^ ^,^ ^42^.

A dimensão “Processo de escolha de entidades e representantes” tem por interesse verificar os mecanismos utilizados para a definição dos representantes nos conselhos de saúde. Para tanto, engloba os indicadores do método de escolha das entidades para comporem o CMS e o processo de escolha do representante pela entidade. O método de escolha do representante foi classificado em três tipologias: presidente da entidade assume a representação; diretoria da entidade indica o representante; e eleição do representante em assembleia ou reunião dos filiados da entidade.

A segunda dimensão, “Relação entre representantes e representados”, diz respeito à forma de acompanhamento da atuação do representante pela entidade e pelos filiados a ela. Busca identificar os mecanismos de *accountability* que envolvem a representação e o interesse dos segmentos representados sobre as ações do CMS.

Por sua vez, na dimensão “Tipos de interesses representados”, busca-se identificar a atuação do conselheiro a partir da representação de interesses assumida. Essa dimensão envolve a defesa de interesses individuais, de interesses corporativos ou de interesses amplos sobre as políticas de saúde.

A quarta dimensão “Critérios para definição de posicionamentos e opiniões” expressa os mecanismos utilizados para a formação da opinião e definição das posturas adotadas pelos representantes. Os resultados dessa dimensão foram classificados em: consciência individual; articulação com outros conselheiros; e orientação da entidade representada. O posicionamento adotado expressa também a força de mobilização social existente nos municípios e como isso influencia na postura dos representantes.

A quinta dimensão, “Prática participativa do representante”, contempla a análise do perfil de atuação a partir dos posicionamentos assumidos nas reuniões. São considerados três perfis de participação: o votante, quando o representante pouco se expressa e a sua atuação restringe-se ao ato de votar; o reivindicativo, que atua na defesa de melhorias de serviços de saúde específicos ou de benefícios para determinados segmentos; e o propositivo/participativo, que assume perfil de atuação colaborativa em busca de soluções para os problemas em curso.

Para o processo de análise, os dados e informações das três técnicas utilizadas foram transformados em registros textuais. Estes foram tratados, organizados e codificados de acordo com a técnica de Análise de Conteúdo Temática ^29^, e analisados a partir das dimensões analíticas utilizadas na matriz teórica.

### Questões éticas

O projeto de pesquisa foi aprovado pelo Comitê de Ética e Pesquisa do Instituto Multidisciplinar em Saúde da Universidade Federal da Bahia (parecer nº 5.406.361). A realização do estudo foi também autorizada pelos três CMS. Todos os participantes assinaram o Termo de Consentimento Livre e Esclarecido.

## Resultados

Os resultados do estudo estão apresentados de acordo com as cinco dimensões apresentadas na matriz de análise. Os excertos das falas representativas de cada dimensão estão dispostos no Quadro 2.

**Quadro 2:** Discursos expressivos sobre representação e representatividade nos conselhos municipais de saúde. Vitória, da Conquista, Guanambi e Urandi, Bahia, Brasil, 2023.

DIMENSÃO	RECORTES DE FALAS
Processo de escolha de entidades e representantes	“*As instituições que representam esses usuários elas fazem uma inscrição da instituição, não da pessoa, e vêm pra eleição. Aqui, as instituições são eleitas e elas vão indicar os nomes para participarem do conselho*” (Entrevista 9, Vitória da Conquista ₋ gestor) “*Pra formar o conselho, eu vejo muito a secretaria convidando algumas associações. Geralmente, na época de formação do conselho, eu não vejo edital. É uma coisa que é formada por convite e só. Durante cada gestão, eles procuram colocar o máximo de pessoas do lado deles, da gestão*” (Entrevista 25, Urandi ₋ usuário) “*Acredito que as associações não têm essa decisão em assembleia pra escolher quem vai ser o representante, não. Geralmente, vai o presidente*” (Entrevista 25, Urandi ₋ usuário) “*Às vezes, aquelas pessoas que querem ficar cativas no conselho, aí ela tem dois anos como titular, dois anos como suplente. Na terceira, ela teria que passar o lugar pra outra, mas ela quer continuar. O que que ela faz? Ela muda de instituição. Vamos supor que ela era de uma representação dos trabalhadores, então ela vai agora pleitear como usuária, pra nunca sair do conselho. Aí você não renova o conselho, você não dá oportunidade para outras pessoas*” (Entrevista 19, Guanambi ₋ prestador) “*Os representantes, a gente sabe que têm problemas dentro do Conselho. Por exemplo, a gente tem profissional de saúde que tá representando usuários, né?! Tem gestor como suplente de trabalhador de saúde. Mas, quando eu tô numa posição de gestão minha visão muda completamente, então eu não posso estar representando um trabalhador, né?*” (Entrevista 4, Vitória da Conquista ₋ gestor) “*Então, quando surge alguém que quer participar, não tem muito o que escolher não, sabe? É aquela coisa, só tem você, vai você mesmo. Dentro da minha comunidade foi assim*” (Entrevista 14, Guanambi ₋ usuário)
Relação entre representantes e representados	“*O grande erro dos conselhos de uma forma geral é o seguinte: a pessoa muitas vezes é representante dele mesmo. Muitas vezes, ele deixa de ser representante de um determinado segmento*” (Entrevista 30, Urandi ₋ gestor) “*A gente não discutia nada com os representados. Para lhe dizer a verdade, a gente nem conversava assim, em relação ao conselho*” (Entrevista 28, Urandi ₋ prestador) “*Eu acho que precisaria ouvir mais as pessoas. O conselheiro também está ouvindo a sociedade pra tentar junto fazer um trabalho melhor*” (Entrevista 23, Urandi ₋ usuário) “*Não com meus pares, mas com a instituição que eu trabalhava, aí tinha. Até porque eles cobravam, ‘ah, vamos levar isso ao conselho’. Então, era a própria instituição*” (Entrevista 15, Guanambi ₋ prestador) “*Sempre levava, mas levava mais a nível de diretoria*” (Entrevista 10, Vitória da Conquista ₋ profissional) “*É marcada a votação e é colocada a pauta. Eu compartilho essa pauta no grupo* [de Whatsapp] *da minha diretoria de associação, não é no grupo geral da associação*”. (Entrevista 20, Guanambi ₋ usuário)
Tipos de interesses representados	“*Eu vejo* [que] *muitos são individualistas, resolve alguma coisa pra eles, mas pro coletivo não vejo muito não*” (Entrevista 25, Urandi ₋ usuário) “*Eu pretendo seguir a carreira política de certa forma, sabe? Num futuro próximo. E através do conselho de saúde eu acho que isso pode tanto ajudar no meu futuro político, como também pode ser uma via de mão dupla*” (Entrevista 20, Guanambi ₋ usuário) “*Olha, esses lugares não é pra ser vitrine, esses lugares é pra se discutir política pública. Mas, também tem gente que tá lá porque quer dizer amanhã na campanha eleitoral: ‘Olha, eu fui conselheiro’. Dá de tudo, né?!*” (Entrevista 2, Vitória da Conquista ₋ usuário) “*O funcionário, o profissional de saúde eles estavam ali, sempre estão fiscalizando e cobrando a parte dos interesses da profissão, né?! Da enfermagem, do salário, do plano de carreira, entendeu?*” (Entrevista 24, Urandi ₋ gestor) “*Muitas vezes, os profissionais de saúde, eles são colocados, são indicados. Na verdade, são contratados, eles não são efetivos. Então, muitas vezes, não têm a devida autonomia para poder questionar dentro do conselho. Por exemplo, um enfermeiro é contratado, muitas vezes ele tem uma posição técnica, que pode, naquele momento, ferir um interesse da gestão. Aí, muitas vezes, o que acontece? Como ele está preso à gestão pública por um contrato, ele não tem a devida autonomia e a independência de questionar adequadamente sobre aquelas demandas. Isso é prejudicial ao sistema de saúde*” (Entrevista 30, Urandi ₋ gestor) “*Aqui, no conselho de saúde, eu deixo de ser ‘Maria’, médica defendendo médico. Eu tenho que defender médico? Tenho. Mas eu tenho que defender o SUS, porque eu estou aqui no controle social do SUS, eu tenho que defender o que é melhor para todos*” (Entrevista 10, Vitória da Conquista ₋ profissional) “*A minha ideologia é o Sistema Único de Saúde, a saúde coletiva, a saúde pública. Então, os meus posicionamentos são sempre em defesa desse conjunto de políticas*” (Entrevista 6, Vitória da Conquista ₋ profissional)
Critérios para definição de posicionamento e opiniões	“*É mais mesmo pelo que eu julgo ser melhor para a comunidade*” (Entrevista 15, Guanambi ₋ prestador) “*Com base no conhecimento, vivência, sabe?! É essa questão de que eu quero para o outro é também o que eu quero pra mim. Então, eu acho que o meu comportamento diante das reuniões é muito um comportamento de empatia, de respeito, de democracia*” (Entrevista 10, Vitória da Conquista ₋ profissional) “*Essa relação, o papel desses profissionais é municiar os usuários com função técnica. É municiar, entendeu?! Então, há essa relação entre nós usuários e profissionais. Nós não temos relação com um governo que se alia muito com o dono, representante de hospital privado, com os médicos*” (Entrevista 2, Vitória da Conquista ₋ usuário) “*Já tem no conselho, digamos, têm 13 ou 14 pessoas ligadas umas às outras. Então, quando um toma decisão, todos tomam. Mas aí, isso que eu te falo, é que às vezes tem aquele voto casado, que a pessoa não sabe em que tá votando e vota né?!*” (Entrevista 4, Vitória da Conquista ₋ gestor) “*Assim, a gente tinha assim, meio uma cupulazinha nossa lá, né?! Dos problemáticos, dos que não deixava nada passar. Qualquer coisinha nós ‘Êpa, isso aí não’*” (Entrevista 18, Guanambi ₋ usuário) “*Cada um por si mesmo. Cada um por si. Todas as reuniões que eu participei*” (Entrevista 26, Urandi ₋ gestor)
Prática participativa do representante	“*Porque muitos conselheiros estão presentes, mas a maioria é mais assim, não estão ativos. Estão presentes, mas não estão ativos*” (Entrevista 22, Urandi ₋ profissional) “*A maioria é bem pacífico. Tem medo de se posicionar e achar que ele é contra o prefeito*” (Entrevista 25, Urandi ₋ usuário) “*Os usuários a gente notava mais observador, não era muito participativo, não*” (Entrevista 24, Urandi ₋ gestor) “*Então esse resultado é no dia a dia, a gente cobrando. Por exemplo, esse PSF que tá fazendo nessa comunidade do Baixio, isso foi uma luta nossa desde antes. Se nós não reivindicar que nossos associados ou que nossos agricultores tenha um atendimento mais perto, então qual é a função nossa?*” (Entrevista 14, Guanambi ₋ usuário) “*Aqui nós bota no canto da parede. Nós quer saber: ‘Veio tanta verba, verba pra o posto de saúde do Vila América, do Bairro Brasil, quanto foi? Onde tá aplicado? Cadê as notas?’ A gente vai lá nos hospitais, fiscaliza, ‘quanto entrou de medicamento e tal?’*” (Entrevista 3, Vitória da Conquista ₋ usuário) “*A gente foi lá, ouviu, escutou, trouxe a informação, pegou o que se tinha de norma e de portaria que trata sobre isso, né?! Sentamos e discutimos e faz-se uma minuta,* (...) *Pronto, encaminha, beleza. Submete ao pleno, abre a discussão, é dado ao governo direito de* (...) *Então, todas as decisões foram tomadas com essa dimensão, talvez isso explique a qualidade do conselho*” (Entrevista 2, Vitória da Conquista ₋ usuário)

### Processo de escolha de entidades e representantes

Evidenciaram-se distintas formas de composição dos CMS nos municípios investigados. Nos municípios de médio e grande porte, mantém-se a rotatividade das entidades que compõem o CMS. Assim, a cada processo de renovação, primeiro realiza-se a escolha das entidades que comporão o pleno e no segundo momento as entidades indicam os representantes. Na cidade de menor porte, o CMS possui uma composição fixa, sendo regido por uma lei municipal que estabelece as entidades que integram o órgão. Identificou-se ainda inexistência de regimento interno no município de pequeno porte, o que mostrou contribuir para a limitação da representatividade.

Os resultados revelaram que em Vitória da Conquista e Guanambi existe um maior interesse por parte das instituições de representação da sociedade civil em compor os respectivos conselhos. Em muitas situações, no processo de renovação do CMS, mais de uma entidade manifesta interesse para representar o mesmo segmento. Assim, é procedida a eleição para escolha da entidade. Em Urandi, identificou-se um processo de desmobilização social e de dificuldades das instituições em indicar representantes para o conselho.

Quanto à escolha dos representantes pelos segmentos, percebeu-se que a eleição não é o mecanismo utilizado para definir os representantes de usuários e trabalhadores. A ascensão destes à cadeira no CMS se dá predominantemente por indicação da diretoria ou por autoindicação do presidente da entidade. Dois fatores mostraram influenciar o predomínio de critérios não eleitorais de escolha dos representantes, um deles foi a falta de interesse dos filiados das entidades em exercer a função de conselheiro de saúde, e o outro foi o interesse dos integrantes das diretorias em manter-se em posição de destaque e em contato próximo com o Executivo municipal.

A baixa rotatividade dos representantes demonstrou influenciar negativamente na representatividade nos CMS. Foi evidenciada a existência de pessoas que se mantém como conselheiro por múltiplos mandatos. O principal artifício utilizado para a manutenção da mesma pessoa na condição de conselheiro foi a mudança de entidade quando do fim do mandato. Nesse sentido, observou-se a formação de um grupo de conselheiros de longa duração que se revezam na representação de entidades diferentes e dificultam a renovação do conselho.

Associado a isso, foi observado o fenômeno de distorção da representação de interesses. Ou seja, as pessoas ao mudar de entidade para se manter no conselho, formalmente passam a representar outra entidade, mas na prática cotidiana exercem a representação de interesses de outro segmento.

### Relação entre representantes e representados

A relação entre representantes e representados é marcada por fragilidades e distanciamentos. Não foram identificadas iniciativas regulares de comunicação e ausculta de demandas. Também não se identificaram mecanismos regulares de prestação de contas ou acompanhamento dos representantes por parte dos representados. Tais achados revelam a atuação isolada do representante e o frágil interesse dos segmentos representados pelas questões tratadas no âmbito dos conselhos.

Nos dois municípios de maior porte, alguns conselheiros mantinham contatos com as diretorias das respectivas entidades. Todavia, tratava-se de relações irregulares para repasse de informações esporádicas. No município de pequeno porte, não se observou qualquer relação dos conselheiros usuários e trabalhadores com os segmentos representados, isso se justifica em razão da baixa mobilização social local.

### Tipos de interesses representados

Houve uma diversidade no tipo de interesse representado. Assim, não se evidenciou um padrão único de interesse assumido pelos conselheiros. A depender do contexto e da pauta em discussão, os conselheiros assumiram postura em defesa de interesses particulares, corporativos e amplos sobre as políticas de saúde.

Sobre os interesses individuais, predominaram a atuação voltada à busca de facilidades para o acesso aos serviços de saúde, especialmente no município de pequeno porte. Evidenciou-se também o exercício da função de conselheiro em busca de projeção político partidária. Nesse sentido, o conselheiro defende interesses diversos, a depender da projeção que possa obter em seus posicionamentos.

Interesses corporativos foram assumidos por representantes dos profissionais de saúde, com destaque para as representações de sindicatos e associações de classe e a representação dos agentes comunitários de saúde. O interesse representado caracterizou-se tipicamente como corporativo, na defesa de direitos trabalhistas e benefícios pecuniários. No âmbito dos CMS investigados, os profissionais de saúde apresentaram um segmento com grande capacidade de influência e com grande poder de articulação junto aos outros segmentos.

Um importante achado do estudo foi a relação entre o tipo de vínculo trabalhista e a autonomia para o exercício da representação. A precariedade dos vínculos de trabalho e a dependência de contratos temporários limitam a representação e constrangem os profissionais a atuar em defesa dos interesses do poder público municipal.

Interesses amplos sobre saúde foram melhor defendidos em Vitória da Conquista. Na cidade de maior porte, as representações dos profissionais e dos usuários não mantinham relações de dependência com o poder local e tinham largo histórico de atuação em movimentos sociais e instituições representativas. Assim, exerciam o mandato representativo de forma independente.

Critérios para definição de posicionamento e opiniões

Predominou entre os conselheiros a consciência individual para a definição de posturas no âmbito das reuniões. Diante do diminuto acompanhamento por parte dos segmentos representados, os conselheiros definem seus posicionamentos a partir da avaliação pessoal do que se constitui como mais adequado para a política de saúde local.

Nos dois maiores municípios, observou-se que existem articulações entre representantes dos trabalhadores e dos usuários com o propósito de definir posicionamentos. Tal movimentação tinha como motivação principal firmar opinião e estabelecer estratégias de contraposição às proposições apresentadas pelos gestores municipais. Essa relação também é viabilizada pela elevada confiança e estima dos representantes dos usuários por um grupo de trabalhadores, que apresentavam em suas trajetórias experiências na gestão municipal e estadual, nos espaços acadêmicos e na defesa do SUS.

### Prática participativa do representante

Quanto à participação em reuniões, as análises demonstraram baixa assiduidade dos representantes dos usuários. Embora os usuários tenham 50% das representações nos CMS, não foi observada uma elevada frequência desse segmento nos diferentes municípios estudados. Tal situação possui ainda maior destaque em Urandi, onde as reuniões são realizadas com quórum predominantemente formado por gestores e trabalhadores para aprovação de pautas burocráticas e institucionais. Nesse contexto, a prática participativa dos conselheiros se caracterizou como votante.

Nos municípios de médio e grande porte, as práticas de participação foram marcadas por perfil reivindicativo e propositivo. Nessas localidades, preponderou a prática da representação questionadora, de reivindicação dos interesses e em defesa do SUS. Identificou-se a associação entre a postura ativa e questionadora com a formação política e social dos representantes.

Também foi percebido que as vivências pregressas de participação comunitária, o vínculo estável de trabalho, a formação para conselheiros de saúde e habilidades pessoais de comunicação concorrem para fundamentar e motivar os representantes à atuação mais abrangente e propositiva nas plenárias dos conselhos.

## Discussão

O estudo revelou fragilidades na representação exercida no âmbito dos CMS. Em todas as dimensões analisadas, constatou-se distorções e insuficiências nos elementos que conferem legitimidade à representação. A representação nos CMS também se mostrou influenciada pelo porte populacional dos municípios. No município de menor população, evidenciou-se fragilidades consistentes para o exercício da representação. Além das insuficiências do processo representativo, os resultados deixam claro as peculiaridades inerentes à representatividade exercida por conselheiros de saúde, que não podem ser avaliadas apenas a partir dos critérios clássicos da representação.

Bispo Júnior & Gerschman ^30^ destacam que a representação nos novos espaços participativos e em instâncias de envolvimento da sociedade civil no seio do Estado é complexa e difere da representação eleitoral clássica. Assim, outros formatos representativos são apresentados como forma de ampliar as experiências participativas. Maia ^8^ sublinha que a representação em instâncias de participação social, muitas vezes, fundamenta-se em legitimidades não eleitorais para a identificação de demandas não percebidas por representantes formalmente eleitos. Saward ^31^ apresenta o conceito de reivindicação representativa para expressar a condição de indivíduos e instituições que reivindicam representar grupos e causas. O autor argumenta que a representação reivindicativa é de grande valor por alargar a compreensão de quem pode agir como representante de causas sociais e o que fundamenta sua atuação.

No âmbito do SUS, a atuação dos representantes sociais exerce grande influência sobre a efetividade dos CMS. Por efetividade participativa, entende-se a qualidade da discussão e deliberação no espaço dos CMS bem como os resultados desencadeados pela atuação dos representantes ^1^ ^,^ ^32^. Assim, o déficit representacional, considerando as diversas dimensões da representação, pode desencadear processos participativos desestimulantes e, consequentemente, fragilização da gestão participativa do SUS.

Nesse sentido, a matriz analítica utilizada no estudo se fundamentou nos diversos aspectos inerentes à representatividade dos conselheiros de saúde, buscando contemplar a natureza multifacetada da representação.

Na dimensão do processo de escolha de entidades e representantes, chama atenção o predomínio de critérios não eleitorais para a escolha dos conselheiros de saúde. Nos três municípios investigados, as práticas mais usuais são de autoindicação do presidente da entidade ou de indicação de um membro por escolha da diretoria. A ausência de eleição dos representantes pelas entidades tem se mostrado uma prática frequente nos conselhos de saúde em outras localidades do Brasil ^32^ ^,^ ^33^. Como agravante, o presente estudo evidenciou que o uso de critérios não eleitorais é potencializado pelo contexto de desmobilização e desinteresse dos membros das instituições em assumir a função de conselheiro.

Outro fator que contribuiu para fragilizar a representação foi a permanência de alguns conselheiros por múltiplos mandatos. Tal prática inibe a renovação dos conselhos e obstaculiza a formação de novas lideranças. Esse fenômeno é denominado de profissionalização da função de conselheiro e também implica em menor diversidade de temas discutidos e interesses representados ^34^. A profissionalização de representantes sociais em fóruns participativos é um fenômeno em destaque na literatura internacional. Holetzek & Holmberg ^2^ salientam que em muitos contextos os representantes de usuários se tornam pessoas altamente qualificadas e instruídas. van de Bovenkamp et al. ^35^ ressaltam que a profissionalização leva a sobrevalorização do saber técnico e, muitas vezes, conduz à exclusão de pessoas consideradas leigas, o que influencia no impacto negativo da qualidade democrática da representação.

A relação estabelecida entre os conselheiros de saúde e os segmentos representados também se mostrou como aspecto crítico da representatividade. Destacam-se nos resultados as práticas do isolamento do representante e a ausência de interação deste com os representados sobre as questões debatidas no CMS. A debilidade desses vínculos abala o processo representativo por diversas perspectivas, uma delas é impossibilitar a ausculta das demandas dos cidadãos. Embora o representante possa ser dotado de boa intencionalidade, é necessário estar atento às expectativas e às necessidades dos segmentos sociais. Para Saward ^31^, diante do distanciamento com as bases, nenhum representante, ainda que empreenda esforços, consegue ser plenamente representativo por meio de suas reivindicações.

Outra implicação da fragilidade dos vínculos com os representados se refere à impossibilidade de acompanhamento da atuação do representante. No mandato representativo, mesmo que se exerça a representação com autonomia, é desejável que os integrantes do segmento possam acompanhar e cobrar determinados posicionamentos do representante ^30^. Holetzek & Holmberg ^2^ adotam o conceito de responsividade para se referir à relação interativa entre os representantes e os cidadãos. Eles destacam que esses mecanismos de interação devem se manter de forma constante para possibilitar uma atuação mais engajada e potencializar os mecanismos de *accountability*. O apoio e a acompanhamento pelos segmentos representados também têm o potencial de fortalecer a atuação do representante, por conceder maior legitimidade para as posturas assumidas.

A dimensão dos tipos de interesses representados é marcada pela heterogeneidade de posicionamentos. De acordo com os resultados, o contexto e o tema em discussão influenciam na postura assumida pelos representantes. Essa diversidade de interesses assumidos é reveladora da complexidade da cultura política no país. Assim, os resultados evidenciaram a existência de posicionamentos políticos na defesa de políticas de saúde abrangentes e do fortalecimento do SUS, assim como demonstraram a persistência de uma cultura política restrita na qual se busca benefícios pessoais no exercício da representação.

De forma similar ao encontrado no presente estudo, o uso da representação para interesses pessoais se faz presente em outras localidades. Em municípios dos estados do Pará ^22^, Santa Catarina ^23^ e Bahia ^32^, também foi identificada a busca por interesses individuais em detrimento de interesses coletivos, especialmente relacionado à facilitação para o uso de serviços de saúde. Tal situação extrapola os aspectos inerentes à participação social no SUS e evidencia a precariedade da assistência ofertada, em face de se recorrer ao status de representante social para viabilizar o acesso aos serviços de saúde.

Ainda sobre os tipos de interesse representados, chama atenção nos resultados como o tipo de vínculo profissional interfere na atuação dos representantes dos trabalhadores e afeta a representatividade no âmbito dos conselhos. Tal achado reforça as evidências dos efeitos danosos da precarização do trabalho em saúde. É amplamente relatado na literatura que a precarização das relações de trabalho conduz a desmotivação, elevada rotatividade e baixa qualidade da assistência prestada ^36^ ^,^ ^37^. Não obstante a esses aspectos, as relações de trabalho instáveis também mostraram contribuir para a fragilização da participação social no SUS. Assim, é possível inferir que a representatividade nos conselhos pode ser fortalecida a partir das políticas de gestão do trabalho e da garantia dos direitos trabalhistas para os profissionais do setor.

Sobre a dimensão critérios para a definição de posicionamentos, os resultados demonstraram que os conselheiros fundamentam a atuação a partir da consciência individual ou a partir da articulação com os demais conselheiros. A debilidade em se fundamentar nas orientações das entidades representadas é naturalmente reflexo da frágil relação estabelecida entre os representantes e representados. Não obstante às implicações desse distanciamento, os posicionamentos a partir do conhecimento experiencial não podem deixar de ser considerados como importante aspecto das instâncias de envolvimento comunitário.

Por conhecimento experiencial entende-se as experiências da vida real que as pessoas têm como usuárias dos serviços ou membros da comunidade, a exemplo de quando acessam os serviços de saúde ou quando lidam com dificuldades estruturais em suas comunidades ^5^. No presente estudo, o conhecimento experiencial mostrou-se pouco valorizado como mecanismo de legitimidade da representação. Em Vitória da Conquista e Guanambi, a articulação entre usuários e profissionais assumiu um caráter instrumental de tecnificação da participação. Como afirma o entrevistado 2 de Vitória da Conquista, essa articulação tem a finalidade de “*municiar os usuários com a função técnica*” (Quadro 2). Assim, ocorre a desvalorização dos elementos comunitários e das experiências vivenciadas nos serviços e a sobrevalorização do caráter técnico da participação, inclusive com o domínio discursivo e a grande capacidade de influência dos profissionais sobre os usuários.

Torna-se importante destacar que as fragilidades do processo representativo não estão localizadas apenas na postura dos conselheiros. Elementos da cultura política do país e as transformações políticas e sociais conduziram ao esmaecimento dos movimentos populares que atuavam na defesa do SUS. Níveis elevados de engajamento comunitário mostraram-se como algo difícil de ser mantido no decorrer dos mais de 30 anos de institucionalização dos conselhos de saúde ^17^. Ventura et al. ^38^ destacam as dificuldades para a consolidação de uma cultura participativa e democrática no Brasil, em face do crescimento de uma cultura política fragmentada e individualista. Nesse sentido, a mobilização e o envolvimento comunitário constituem-se como importantes desafios para a participação no SUS, o que interfere na representatividade dos conselheiros de saúde.

Em relação à dimensão prática participativa, o porte populacional mostrou interferir na atuação dos representantes. O perfil votante e pouco propositivo foi predominante entre os conselheiros de Urandi. Isso pode ser explicado pela diminuta mobilização social e o clientelismo ainda frequentes em municípios de menor população. Esse contexto favorece a dominação da agenda pelos governantes, que têm interesses restritos de aprovação de contas e dos projetos de interesse da administração ^39^. Rojatz et al. ^40^ apontam o perigo da influência do governo na autenticidade dos ambientes participativos em saúde, capaz de comprometer a credibilidade representativa desses espaços decisórios.

As práticas participativas reivindicativa e propositiva estiveram mais presentes em Guanambi e Vitória da Conquista, o que se mostrou associado à estabilidade profissional, experiências participativas anteriores e formação para o controle social. A relação entre representatividade dos conselhos e o porte populacional dos municípios foi também identificada em municípios de Santa Catarina, que apresentaram melhores resultados em cidades com população mais numerosa ^41^. Posturas reivindicativas e propositivas são resultantes da maior autonomia dos representantes ^42^. Em contextos em que predominam a dependência da população à vontade do poder público local e os direitos sociais e de saúde são confundidos com benesses clientelistas, a autonomia dos representantes é fragilizada e o conselheiro acaba por assumir uma prática votante sob a influência do gestor municipal.

## Considerações finais

A representação apresenta-se como aspecto crítico e elemento essencial para garantir maior efetividade dos conselhos de saúde. Nos municípios estudados, a representatividade mostra-se fragilizada conforme os resultados referentes às cinco dimensões que integram a matriz analítica utilizada. Destacaram-se mecanismos de escolha pouco representativos, isolamento dos representantes, busca de interesses individuais sobre os interesses coletivos e tecnificação da participação. Também foi evidenciado que a representação é ainda mais fragilizada na cidade de menor porte, com menor autonomia dos representantes e maior prática clientelista pela gestão municipal.

Fortalecer a representação e a representatividade dos CMS perpassa por transformações estruturais, organizativas e da cultura política. Nesse sentido, ressalta-se a necessidade de desenvolver mecanismos de aproximação entre os representantes e os segmentos representados. Também salienta-se a importância da valorização do conhecimento experiencial como elemento capaz de conceder legitimidade à representação. Assim, desenvolver processos formativos com ênfase nos elementos comunitários e na dimensão política da participação constitui-se como ação estratégica para o alcance de melhores resultados na representação dos CMS.
